# Sensory Acceptability and Sensory Profiles of Flavoured Foods for Special Medical Purposes: A Quantitative Descriptive Analysis

**DOI:** 10.3390/jcm15062188

**Published:** 2026-03-13

**Authors:** Agata Kiciak, Wiktoria Staśkiewicz-Bartecka, Natalia Kuczka, Małgorzata Słoma-Krześlak, Kommi Kalpana, Marek Kardas

**Affiliations:** 1 Department of Food Technology and Quality Assessment, Department of Dietetics, Faculty of Public Health in Bytom, Medical University of Silesia in Katowice, ul. Jordana 19, 41-808 Zabrze, Poland; wstaskiewicz@sum.edu.pl (W.S.-B.); mkardas@sum.edu.pl (M.K.); 2 Doctoral School, Medical University of Silesia, 40-055 Katowice, Poland; d201362@365.sum.edu.pl; 3 Department Human Nutrition, Department of Dietetics, Faculty of Public Health in Bytom, Medical University of Silesia in Katowice, ul. Jordana 19, 41-808 Zabrze, Poland; msloma-krzeslak@sum.edu.pl; 4 Faculty of Allied Health Sciences, Manav Rachna International Institute of Research and Studies, Sector 43, Aravalli Hills, Delhi—Surajkund Road, Faridabad 121004, India; kommikalpana80@gmail.com

**Keywords:** food for special medical purposes, FSMP, sensory analysis, sensory acceptability, QDA, malnutrition

## Abstract

**Background/Objectives** Foods for special medical purposes are an important component of nutritional management in patients at risk of malnutrition or already affected by it. The aim of the study was to evaluate the sensory properties and sensory acceptability of selected powdered foods for special medical purposes (FSMPs) from a single manufacturer with different flavour variants using quantitative descriptive analysis. **Methods**: The study was conducted under laboratory conditions in accordance with the PN-EN ISO 8589:2010 standard. A semi-trained panel of 49 participants took part in the sensory evaluation. Five powdered FSMP products with comparable nutritional composition and different flavour variants were analyzed. Quantitative descriptive analysis (QDA) was applied using a 10-point rating scale, along with a ranking method to assess consumer preferences. Statistical analysis was performed using Welch’s one-way analysis of variance followed by post hoc tests, with the level of significance set at *p* < 0.05. **Results**: Statistically significant differences were observed between the analyzed variants for most of the assessed sensory attributes. Preparations with white chocolate and raspberry, strawberry, and vanilla flavours showed the highest sensory acceptability, characterized by a harmonious taste, creamy texture, and low intensity of undesirable sensory attributes. The neutral variant received the lowest overall ratings. The coffee-flavoured product was distinguished by a high aroma intensity but also by a greater intensity of attributes negatively affecting sensory acceptability. **Conclusions**: The findings indicate that flavour plays a significant role in determining the sensory acceptability of FSMP products. Considering sensory characteristics in the development and selection of FSMP preparations may increase their consumption and enhance the effectiveness of nutritional interventions in clinical practice.

## 1. Introduction

Malnutrition is a significant factor that worsens prognosis in the course of many chronic diseases. This problem may affect any patient, particularly older adults, who are more vulnerable to disturbances in nutritional status. In the treatment of malnutrition, both in hospital settings and in outpatient care, foods for special medical purposes are used [1].

This category includes, among others, oral preparations in liquid or powder form, which can be consumed on their own or added to traditional meals. The inclusion of foods for special medical purposes should be considered in patients who, due to health limitations, are unable to meet their energy and nutrient requirements through their usual diet alone, as well as in individuals who are malnourished or at risk of malnutrition, according to diagnostic criteria. When selecting the appropriate preparation, the patient’s clinical condition, taste preferences, and the cost of therapy should be taken into account, especially since most of the available products in Poland are not reimbursable. It is worth emphasizing that effective nutritional intervention should be based not only on the use of foods for special medical purposes, but also on providing patients with access to dietary consultations already at the primary health care stage [1,2,3,4,5].

Foods for special medical purposes (FSMPs, ang. foods for special medical purposes) are an important part of nutritional therapy for patients with impaired ability to ingest, digest, or absorb food, and for people requiring precise dietary support under medical supervision. FSMPs are dietary preparations designed for patients whose health condition prevents them from meeting their full energy and nutritional requirements, which are often increased during the course of their illness, through a traditional diet. This applies, among others, to people with chronic diseases, cancer, metabolic disorders (e.g., diabetes), or neurological conditions. These preparations are also used in the perioperative period and during convalescence, supporting the healing process and recovery of fitness [5,6,7].

FSMP formulas are usually liquid or semi-liquid, which makes them easier to ad-minister to patients with limited chewing and swallowing abilities. A key feature of these preparations is their flexibility; they can be used both as a supplement to the daily diet and, in appropriate clinical indications, as a full substitute for it. This makes FSMP a tool for maintaining or improving nutritional status in situations where the intake or utilization of conventional meals is insufficient [5].

In the European Union, FSMPs are defined and regulated by legal provisions and guidelines that specify requirements for composition, labelling, and scientific evidence necessary to justify the clinical applications of these products [6,8]. Growing interest in foods for special medical purposes on the part of manufacturers and the medical community has led to intensified research into the safety, bioavailability, and clinical efficacy of oral nutritional supplements, both in liquid and powder form [9].

Systematic reviews and analyses provide mixed but increasingly robust evidence that appropriately selected oral energy–protein supplements can improve anthropometric parameters and selected nutritional indicators in patients at risk of malnutrition. A key factor in the effectiveness of FSMPs is their acceptance in terms of organoleptic characteristics (taste, smell, and texture), which directly determines the degree to which patients comply with recommendations for long-term supplementation. Studies comparing the different flavours of oral supplements (e.g., chocolate, vanilla, strawberry, coffee) indicate that flavour preferences vary depending on the population and clinical context, and that the choice of flavour can increase product consumption and thus its therapeutic effectiveness. A practical convenience that promotes greater flexibility in the use of FSMPs is also the wide range of flavour options, including a neutral option and powder forms, allowing for the preparation of a drink with any flavour [10].

In light of the above findings, it is reasonable to consider specific examples of ready-made foods for special medical purposes (FSMPs) available on the market. Selected powdered FSMPs from a single manufacturer, available in various flavours, such as white chocolate with raspberry, coffee, neutral, strawberry, and vanilla, illustrate the flexible use of FSMPs. Their composition, which includes a set of vitamins and minerals as well as sources of protein and carbohydrates, combined with their powder form, allows them to be used both as a dietary supplement and as partial or total oral nutrition in specific clinical indications, in compliance with applicable guidelines and under the supervision of medical personnel. At the same time, it should be emphasized that for many brands and flavour variants, there is a lack of large-scale, independent randomized studies, which indicates the need for further research to evaluate the long-term efficacy and tolerance of individual FSMP preparations [7,11].

The aim of this study was to evaluate the sensory properties, sensory profiles, and overall sensory acceptability of five flavour variants of powdered FSMPs with comparable nutritional compositions using quantitative descriptive analysis (QDA).

## 2. Materials and Methods

### 2.1. Test Procedure

The tests were conducted in the sensory analysis laboratory of the Department of Dietetics, Medical University of Silesia in Katowice, Faculty of Public Health in Bytom. The laboratory where the tests were carried out met the requirements and assumptions of the PN-EN ISO 8589:2010 Sensory Analysis—General guidelines for the design of sensory analysis laboratories [12].

Sensory analysis of products in the analytical part of the study was carried out by teams of 5 or 6 people. Sensory evaluation was conducted in a dedicated sensory analysis laboratory designed and equipped in accordance with the PN-EN ISO 8589:2010 standard, ensuring controlled environmental conditions (lighting, temperature, ventilation, and isolation of assessors). The study was conducted from March to May 2025. QR codes were used to provide access to an electronic evaluation form created using Google Forms (Google LLC, Mountain View, CA, USA). The platform enabled anonymous data collection and direct electronic recording of responses.

### 2.2. Study Participants

Participants were undergraduate and graduate students majoring in Dietetics at the Silesian Medical University in Katowice. The sample was selected purposively and included individuals without declared smell or taste disorders, non-smokers, and those without food allergies or intolerances to the ingredients of the evaluated preparations. Participation in the study was voluntary, and all participants were informed about the purpose and procedure of the study.

Before proceeding with the actual sensory evaluation, students were trained by a team of experts in sensory analysis, in accordance with the guidelines of the PN-ISO 22935-1 standard. The training was aimed at standardizing the perception and interpretation of the evaluated sensory characteristics, as well as limiting the impact of subjective individual differences between evaluators. The training process included the following: a theoretical part, during which participants learned the basics of sensory analysis, the principles of quantitative descriptive analysis (QDA), the definitions of the evaluated attributes (appearance, smell, taste, texture, and consistency), and the principles of working in a sensory analysis laboratory; a practical part, consisting of exercises in assessing the intensity of selected sensory characteristics on reference samples, which enabled the calibration of assessments and a better understanding of the assessment scale; instruction on the use of a 10-point scoring scale, including the interpretation of extreme values (0—no attribute, 10—very high intensity of the attribute) and the principles of consistent scoring; and a discussion of the principles of taste neutralization between samples and the importance of the order of scoring, environmental conditions, and concentration during the test. After completing the training, participants demonstrated the ability to consciously and consistently evaluate sensory characteristics, which allowed them to be classified as a semi-trained panel suitable for conducting analytical and consumer sensory tests.

### 2.3. Organoleptic Evaluation of Products

The research material consisted of five powdered FSMPs manufactured by the same company (Olimp Laboratories, Dębica, Poland). Four products belonged to the Nutramil product line, while one product (Immuven, coffee flavour) was marketed under a different trade name. The analyzed variants included the following flavours: white chocolate with raspberry, coffee, neutral, strawberry, and vanilla.

All preparations were intended for comparable clinical use and had broadly similar nutritional profiles, differing primarily in their flavour characteristics. The inclusion of the coffee-flavoured Immuven variant enabled comparison of a distinct flavour profile within products from the same manufacturer. This design allowed for the assessment of the influence of flavour variation on sensory perception and participant preferences.

The selected food products prepared for analysis are presented in Table 1.

The selected FSMP products used in the sensory evaluation differed in nutritional composition depending on the type of product and its clinical purpose. These products had a similar energy and vitamin–mineral profile, but differed in protein content and additional functional ingredients tailored to the specific nutritional needs of patients. The higher protein content in selected variants allowed for partial or complete coverage of the daily requirement in people requiring protein supplementation. A detailed analysis of the composition of individual variants is presented in Table 2.

During the research, the intensity of selected performance characteristics was assessed, such as:

1. Appearance (visual assessment of the product): colour uniformity, colour intensity, clarity (for liquid preparations), and presence of sediment or stratification.
2. Smell (aroma assessment): intensity of the scent, naturalness of the aroma, and presence of undesirable notes (e.g., foreign, chemical).
3. Taste: intensity of the basic taste (e.g., sweet, salty), harmony of flavour (are the ingredients well balanced?), presence of undesirable notes (e.g., bitter, metallic), purity of taste, and intensity of aftertaste.
4. Texture and consistency: smoothness, creaminess, and presence of lumps/graininess.

#### 2.3.1. Evaluation of Medical Preparations in the Category of Foods for Special Medical Purposes Using a Scoring Method

The evaluators received QR codes, which had to be scanned in order to access the electronic form used for the sensory evaluation of the samples. The form contained in-formation on all the characteristics being evaluated.

A proprietary 10-point intensity rating card (0—attribute not perceptible; 10—extremely high intensity) was used, based on Polish Standard PN-ISO 22935-1: Sensory Analysis—Part 1: General guidelines for the recruitment, selection, training, and monitoring of assessors [13]. The scale was applied in accordance with the principles of quantitative descriptive analysis (QDA), enabling the assessment of the intensity of specific sensory attributes rather than hedonic liking alone. Unlike the 9-point hedonic scale commonly used in consumer acceptance studies, the present study focused primarily on analytical sensory evaluation conducted by a semi-trained panel. Therefore, the use of a 10-point intensity scale allowed for more precise differentiation of attribute intensity and was considered methodologically appropriate.

The following sensory parameters were evaluated: appearance, smell, taste, texture, and consistency. This method was selected as it provided an appropriate level of complexity tailored to the skills and experience of the participating students.

The method of preparing samples for the evaluation of vegetarian products and their proprietary counterparts is presented in Table 3.

Each participant in the study received a set of five coded samples. Each sample in a given set represented a specific weight and belonged to a selected group of FSMP medical preparations. In addition, respondents received a bottle of still mineral water and sugar-free rusks to cleanse their taste buds before each evaluation.

All samples were coded with random three-digit numerical codes to prevent product identification. The order of sample presentation was randomized across participants to minimize potential order and carry-over effects.

#### 2.3.2. Evaluation of Medical Preparations in the Category of Foods for Special Medical Purposes Using the Ranking Method

Another element of the study was the sensory evaluation of selected products using the ranking method. Participants were asked to assign each sample a rank (number 1—the most preferred sample, number 5—the least preferred sample). Each project participant received a set consisting of five coded samples. Each sample in a given set represented a specific weight and belonged to a selected group of medical preparations. In addition, respondents received a bottle of still mineral water and sugar free rusks to cleanse their taste buds before each evaluation.

Each product was evaluated once by each participant during a single sensory session.

In total, fifteen sensory attributes were included in the final statistical analysis (Table 4), covering appearance-related (colour intensity, colour uniformity, clarity, and presence of sediment), aroma-related (intensity of scent and natural aroma), taste-related (purity of taste, harmony of flavour, intensity of basic taste, intensity of aftertaste, presence of undesirable notes including foreign/chemical and bitter/metallic notes), and texture-related characteristics (smoothness, creaminess and presence of lumps/graininess).

### 2.4. Statistical Analysis

The obtained data were analyzed using Statistica v.13.3 (StatSoft Polska, Kraków, Poland) and R v.4.0.0 (2020) under the GNU GPL licence (The R Foundation for Statistical Computing, Vienna, Austria).

Descriptive analysis of the results was performed by calculating arithmetic means ($\bar{x}$), standard deviations (SDs), and standard errors of the mean (SEs), which allowed for the assessment of the level of variability and the degree of diversity in the intensity of individual sensory attributes of the evaluated preparations.

In order to verify compliance with the assumptions of variance analysis, an assessment of variance homogeneity was performed using Levene’s test, which showed a lack of homogeneity of variance between the compared groups. Therefore, Welch’s one-way ANOVA, recommended in cases of violation of the assumption of homogeneity of variance, was used for further statistical analysis. To identify statistically significant differences between individual product variants, Tukey’s post hoc test was used, allowing for detailed pairwise comparisons. A value of *p* < 0.05 was adopted as the level of statistical significance.

In addition, radar charts were developed for a multidimensional presentation and comparison of the sensory profiles of all five tested preparations. This format enabled the simultaneous assessment of the intensity of individual sensory attributes and the visual identification of qualitative differences between products, such as dominant flavour notes, sweetness level, colour intensity, textural properties, and overall acceptability.

## 3. Results

### 3.1. Sensory Evaluation of Products

A total of 49 participants were included in the final sensory evaluation. Each flavour variant was evaluated once by each participant, resulting in 49 independent evaluations per product.

Preliminary data analysis revealed clear differences in sensory profiles among the tested foods for special medical purposes. The white chocolate and raspberry, strawberry, and vanilla variants received the highest average scores for most key sensory parameters, including purity and harmony of flavour, smoothness, creaminess, and intensity of basic taste. This indicates a more balanced and favourable overall sensory profile. In particular, the white chocolate and raspberry variant stood out with high scores for flavour purity and harmony, combined with a low intensity of undesirable attributes, suggesting high sensory acceptability among evaluators (Table 5).

The neutral variant received the lowest average scores across most key sensory categories, especially in terms of flavour harmony (1.43), intensity of basic taste (1.96), aroma intensity (2.69), and flavour purity (2.41). These results indicate a significantly less favourable sensory profile compared to the other variants. This may suggest the need for reformulation, particularly with regard to enhancing flavour intensity and improving overall aroma and taste quality (Table 5).

The coffee-flavoured variant demonstrated a distinct sensory pattern. It achieved the highest mean scores for aroma intensity (8.04) and natural aroma (6.14), indicating a strongly perceptible aromatic profile. However, it was also characterized by the highest intensity of undesirable bitter and metallic notes (5.20), as well as greater graininess and presence of sediment (6.78). This combination suggests that despite pronounced aromatic characteristics, overall sensory perception may be negatively influenced by structural and taste-related defects (Table 5).

The strawberry and vanilla variants received consistently high ratings in key categories such as creaminess, smoothness, flavour intensity, and colour uniformity. The vanilla variant, in particular, presented a well-balanced profile with a low intensity of undesirable attributes, indicating high sensory homogeneity and acceptability (Table 5).

Overall, the analysis of sensory results indicates that the white chocolate and raspberry, strawberry, and vanilla variants exhibited the most favourable and balanced sensory profiles among the evaluated products. In contrast, the neutral variant received significantly lower ratings in most key attributes, indicating the least favourable sensory profile within the tested group. Although the coffee-flavoured variant demonstrated high aroma intensity, the presence of taste and texture-related defects contributed to a less consistent overall sensory profile, suggesting potential areas for formulation improvement.

### 3.2. Analysis of Sensory Diversity of FSMP Products

A one-way analysis of variance using Welch’s test demonstrated statistically significant differences among the tested products for most of the evaluated sensory attributes.

The strongest main effects were observed for harmony of flavour (F = 31.912; *p* < 0.001), intensity of basic taste (F = 36.955; *p* < 0.001), and aroma intensity (F = 28.793; *p* < 0.001). These findings indicate substantial differences in flavour and aroma perception between the analyzed variants (Table 6).

Statistically significant differences were also identified for flavour purity (F = 9.637; *p* < 0.001), intensity of aftertaste (F = 8.916; *p* < 0.001), colour uniformity (F = 14.415; *p* < 0.001), and natural aroma (F = 15.795; *p* < 0.001), as well as for texture-related attributes such as clarity (F = 4.848; *p* = 0.001), creaminess (F = 2.749; *p* = 0.031), and presence of lumps or graininess (F = 2.778; *p* = 0.030). These results indicate that the products differed not only in terms of flavour and aroma characteristics, but also with respect to structural and visual quality (Table 6).

No statistically significant differences were observed for colour intensity (F = 2.000; *p* = 0.099) or for the general presence of undesirable off-flavours (F = 1.977; *p* = 0.102). This suggests that the evaluators did not clearly differentiate between variants in these specific attributes (Table 6).

At the same time, significant differences were found for specific undesirable attributes, including bitter or metallic notes (F = 5.711; *p* < 0.001) and the presence of sediment or delamination (F = 3.334; *p* = 0.013), indicating variability in the technological quality and physical stability of the analyzed preparations (Table 6).

Pairwise comparisons between individual products demonstrated that the neutral variant received significantly lower ratings for most key sensory attributes, particularly those related to flavour and aroma. In contrast, the coffee-flavoured variant was characterized by the highest aroma intensity and natural aroma; however, it also exhibited a significantly greater presence of sensory defects, including bitter and metallic notes, increased sediment formation, and reduced clarity.

The white chocolate and raspberry, strawberry, and vanilla variants achieved the most balanced and consistently high scores, with a low intensity of undesirable attributes. Post hoc analysis confirmed that these variants represented the highest overall sensory quality, whereas the neutral and coffee-flavoured variants displayed statistically significant undesirable characteristics.

The detailed comparison presented in Table 7 revealed numerous statistically significant differences between the evaluated variants (*p* < 0.05). The neutral variant received the lowest scores for key sensory parameters, including flavour purity, harmony of flavour, intensity of basic taste, and aroma intensity. These differences were confirmed by multiple significant pairwise comparisons (e.g., white chocolate and raspberry vs. neutral, *p* < 0.001; coffee vs. neutral, *p* < 0.001; strawberry vs. neutral, *p* < 0.001), indicating a clearly less favourable sensory profile in terms of flavour and aroma quality.

Although the coffee-flavoured variant demonstrated the highest aroma intensity, it was also associated with a greater presence of undesirable bitter and metallic notes, increased graininess, and a higher tendency to form sediment. Significant intergroup differences (e.g., coffee vs. vanilla for metallic notes, *p* = 0.012; coffee vs. strawberry for sediment, *p* = 0.030) further support the conclusion that this variant presented a less consistent sensory profile.

The most favourable and balanced sensory profiles were observed for the white chocolate and raspberry and vanilla variants, which received high ratings for flavour purity and harmony, combined with a low presence of structural and taste-related defects. The absence of statistically significant differences in several comparisons (e.g., white chocolate and raspberry vs. vanilla; *p* = 0.978 for colour uniformity) suggests that these variants are sensorily stable and well accepted (Table 7).

### 3.3. Graphical Presentation of FSMP Product Sensory Profiles

**Figure 1:**
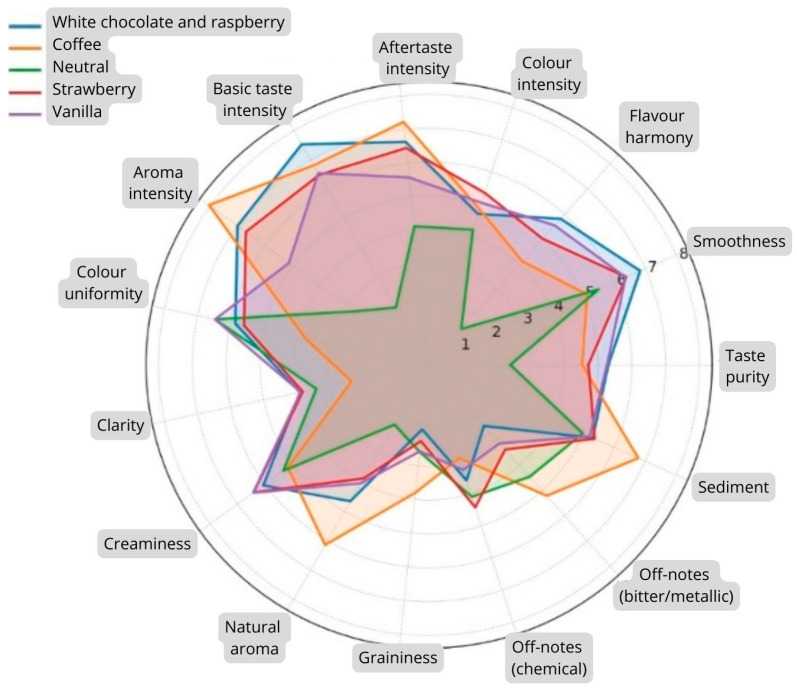
Radar chart presenting the sensory profiles of five flavour variants of powdered foods for special medical purposes (FSMPs) evaluated using quantitative descriptive analysis (QDA). The numbers on the radial scale represent mean intensity scores obtained using a 1–9 structured sensory scale, where higher values indicate greater perceived intensity of a given attribute.

Analysis of the sensory profiles presented in the radar charts allows for detailed characterization of the quality of each of the tested products based on the quantitative descriptive analysis (QDA) method. The overall sensory profile of all five products is presented in Figure 1.

The sensory profile of the white chocolate and raspberry variant demonstrates a well-balanced set of attributes, characterized by a high intensity of desirable sensory characteristics and low levels of undesirable attributes. The product is distinguished by high smoothness, strong harmony of flavour, and pronounced intensity of basic taste and aftertaste, as well as elevated aroma intensity. Negative attributes, including graininess, undesirable flavour notes, and sediment formation, remained at low levels, indicating good technological stability and sensory consistency. The combination of these characteristics suggests high sensory acceptability and a well-balanced overall profile compared to the other evaluated variants.

The coffee-flavoured variant exhibited a clear dominance of aroma intensity, reaching one of the highest values among all assessed attributes. The product was also characterized by a moderate intensity of basic taste and average levels of smoothness and harmony of flavour, suggesting a generally acceptable level of desirable sensory characteristics. At the same time, elevated values were observed for undesirable attributes, including bitter and metallic notes, increased graininess, and reduced clarity compared to the other variants. These characteristics indicate a less homogeneous sensory structure and the presence of defects that may negatively influence perceived product quality. Overall, although the coffee-flavoured variant presented a distinctive and intense aromatic profile, it was more prone to flavour- and texture-related defects, which may reduce its overall sensory acceptability.

The neutral variant was characterized by low values for key flavour-related attributes, including flavour purity, harmony of flavour, intensity of aftertaste, and intensity of basic taste. This indicates limited flavour distinctiveness and insufficient balance among the components of the flavour profile. The neutral variant also exhibited higher intensity of undesirable attributes, such as foreign or chemical notes, bitter and metallic notes, and increased sediment presence. These findings suggest a less stable sensory structure and the presence of defects that may negatively affect overall quality perception. Although relatively higher values were observed for smoothness and colour uniformity, these characteristics did not compensate for the low ratings of flavour-related attributes and the elevated intensity of undesirable notes. Overall, the profile indicates a heterogeneous and poorly balanced sensory structure, which may limit acceptability.

The strawberry variant demonstrated high intensity for most desirable attributes, resulting in a consistent and well-balanced sensory character. Particularly high values were observed for smoothness, aroma intensity, intensity of basic taste, and intensity of aftertaste, suggesting a well-developed and harmonious flavour and aroma profile. Appearance-related attributes, including colour uniformity and clarity, were rated at moderate levels, indicating generally appropriate, though not fully homogeneous, visual quality. The low intensity of undesirable characteristics, such as graininess, foreign notes, and bitter or metallic notes, reflects good sensory stability and the absence of pronounced defects. Overall, this variant exhibited good sensory quality and a balanced profile.

The vanilla variant also presented a well-balanced set of desirable attributes, including high smoothness, strong harmony of flavour, elevated intensity of basic taste, and pronounced creaminess. High colour uniformity suggests appropriate mixing of ingredients and good physical stability. Undesirable attributes, such as graininess, foreign notes, and bitter or metallic notes, were rated at low levels, indicating the absence of significant sensory defects. Overall, this variant exhibited a favourable and consistent sensory structure.

Analysis of the sensory profiles enables clear differentiation of the evaluated products with respect to flavour, aroma, and texture characteristics. The white chocolate and raspberry, strawberry, and vanilla variants demonstrated a balanced combination of desirable attributes, accompanied by a low intensity of undesirable characteristics, indicating stable and favourable sensory profiles. The coffee-flavoured variant exhibited a more heterogeneous structure, with coexisting positive aroma characteristics and increased undesirable notes. In contrast, the neutral variant showed the lowest intensity of desirable attributes and a relatively higher presence of defects, resulting in the least favourable overall sensory profile among the tested products.

All radar charts illustrating the detailed sensory profiles of the individual variants have been included in the Supplementary Materials to improve the clarity and readability of the main manuscript.

## 4. Discussion

Foods for special medical purposes constitute a separate group of food products developed for patients with diagnosed conditions that limit their ability to properly consume, digest, absorb, metabolize, or excrete nutrients contained in a standard diet. These preparations are also aimed at people with specific nutritional requirements resulting from their clinical condition, which cannot be effectively met solely by modifying their traditional diet [8].

An increasing number of patients worldwide require specialized nutritional support during chronic diseases, acute conditions, and periods of convalescence. In such situations, the daily diet often proves insufficient to meet energy and nutrient requirements, especially in people at risk of malnutrition or already malnourished [4,7]. In response to these needs, foods for special medical purposes, which are an important part of nutritional therapy, are becoming increasingly common. These preparations are used both to prevent the worsening of malnutrition and to treat nutritional disorders resulting from chronic, cancerous, metabolic, and neurological diseases, as well as surgical procedures. FSMPs provide appropriately balanced nutrients in a form tailored to the patient’s capabilities and needs, supporting the maintenance or restoration of normal body functions and improving treatment outcomes [17,18].

The results of this study demonstrate clear differences between the flavour variants of foods for special medical purposes in terms of sensory acceptability. Variants characterized by a more balanced flavour and aroma profile—namely white chocolate and raspberry, strawberry, and vanilla—received significantly higher ratings for both the intensity of desirable attributes (flavour, aroma, smoothness) and the low intensity of undesirable characteristics compared to the neutral and coffee-flavoured variants.

These findings suggest that sensory characteristics play a crucial role in product evaluation and may substantially influence overall acceptability in the context of clinical use.

Sensory acceptance of oral preparations is one of the key factors determining their intake and compliance with dietary recommendations. The literature emphasizes that sensory barriers, such as unpleasant taste, smell, or texture, significantly reduce patients’ willingness to regularly consume oral preparations, which can lead to reduced compliance with nutritional therapy and poorer clinical outcomes [19,20].

In a study by Vidal et al. [21] evaluating the sensory acceptability of oral nutritional supplements used in malnourished patients, it was shown that preparations with a sweet taste profile, especially those with a chocolate flavour, were significantly better accepted than vanilla and strawberry variants. Higher sensory acceptability translated into better product tolerance and a greater willingness to consume it again.

Furthermore, as indicated by the literature review by Lester et al. [19] and Cawood et al. [22], differences in the sensory acceptance of oral supplements can significantly affect the amount of the preparation actually consumed and, consequently, the effectiveness of nutritional intervention, especially in populations of patients with a limited ability to eat. The results obtained in this study, indicating significantly higher sensory acceptability of the SBCIM, ST, and SW variants, are consistent with the latest literature reports emphasizing the key role of taste and smell characteristics in the perception of oral nutritional preparations.

A study by Delompré et al. [23] showed that the presence of bitter, metallic, and tart notes is one of the main factors reducing the acceptability of nutritional supplements, even when their nutritional value is adequate. The authors emphasized that sensory abnormalities can significantly impair the overall perception of the quality of a preparation, which is directly reflected in the results of this study, especially with regard to the coffee variant, which, despite its high aroma intensity, was characterized by a distinct presence of undesirable notes and a lower overall rating.

The convergence of these observations is also confirmed by the results of a review by Kang and Park [5], who pointed out that the sensory appeal of FSMPs remains one of the key challenges in their effective clinical application. The authors emphasize that even preparations with documented nutritional efficacy may be consumed in insufficient quantities if their sensory profile does not match patient preferences. In this study, significant differences between flavour variants confirm this relationship, indicating that harmonious taste and reduced sensory defects promote higher acceptance of FSMP products.

It is worth comparing the obtained results to the latest randomized clinical trial con-ducted by Iglesias Hernández et al. [24], which assessed the tolerance and satisfaction of patients using high-energy oral nutritional supplements. The authors demonstrated that good tolerance and positive evaluation of the preparation promoted its regular consumption, which indirectly translated into improved nutritional status parameters. Although this study did not include a quantitative sensory analysis, it emphasized the importance of product acceptability as a factor supporting the effectiveness of nutritional intervention. These results are consistent with the observations of the present study, in which the variants with the highest sensory ratings may potentially promote better adherence to nutritional recommendations.

The importance of the acceptability of oral preparations is further confirmed by the results of a systematic review by Iwańska et al. [25], which showed that high patient compliance with oral nutritional supplements is possible, especially when the preparations are well tolerated and accepted. The authors suggest that although sensory acceptability is not always directly assessed, it plays an important role in long-term adherence to nutritional recommendations, which is confirmed by the results of this study.

When comparing our results with current literature data, it should be noted that both in this study and in the cited publications, the most important factors for the acceptability of FSMP preparations were taste and aroma characteristics, such as taste harmony, basic taste intensity, and the presence of undesirable notes [5,23,24,25]. The convergence of these observations confirms the validity of considering sensory aspects as one of the key elements in the design and optimization of recipes for foods for special medical purposes.

The SN (neutral) variant received the lowest scores in most of the sensory categories evaluated, particularly in terms of flavour intensity and harmony and aroma intensity. Although neutral preparations are often designed for broad clinical use and compatibility with other food products, numerous studies indicate that their sensory acceptability can be limited, especially when consumed without flavour additives, which is associated with the dominance of bitterness, astringency, or a metallic aftertaste derived from the active ingredients [18,26]. The low flavour intensity and the presence of foreign notes may lead to reduced consumption of the preparation and thus to a decrease in the effectiveness of the nutritional intervention [18].

Product SK (coffee) stood out for its highest aroma intensity and natural flavour, but at the same time it was characterized by a significantly higher intensity of undesirable characteristics, such as bitterness, metallic notes, graininess, and the presence of sediment. Similar observations have been reported in other studies, which have shown that an intense aroma profile, including a coffee aroma, does not always correlate with high sensory acceptance if it is accompanied by taste or texture defects, such as excessive bitterness or undesirable mouthfeel. This may suggest that aroma intensity should be balanced by an appropriate product structure and purity of the flavour profile [26,27].

Another aspect is the impact of sensory methodology on product evaluation. In medical food research, it is important to take into account the specific characteristics of the study group, such as age, health status, or changes in sensory perception (e.g., taste and smell). The literature indicates that older people and patients with chronic diseases may have reduced sensory perception, which requires the use of appropriately adapted research methods and sensory analysis tools [28].

A study using quantitative descriptive analysis (QDA) is a reliable method for differentiating sensory profiles, but future studies should include target groups, including clinical patients, to reflect real consumer and therapeutic behaviours. The clinical implications of the results obtained are significant, as improving the sensory acceptance of foods for special medical purposes (FSMPs) may directly translate into better compliance with dietary recommendations and, consequently, improved nutritional status and treatment outcomes [29].

Although the study was conducted primarily among healthy students, in clinical practice, regular consumption of oral nutritional supplements plays an important role in effective nutritional therapy, contributing to improved nutritional status, weight gain, and reduced risk of complications resulting from malnutrition [29]. The results obtained therefore indicate the need to design FSMP preparations taking into account diverse flavour profiles and organoleptic characteristics that better correspond to the preferences and needs of patients [27].

The results of the variance analysis showed that the greatest differences between the evaluated products concerned taste and aroma characteristics, in particular taste harmony, basic taste intensity, and aroma intensity. These observations are consistent with reports in the literature, which emphasize that these parameters are the ones that most affect the subjective assessment of product quality and FSMP consumer preferences [26]. An important methodological element of the study was the use of radar charts, which enabled a clear, multidimensional presentation of the sensory profiles of individual variants. This type of visualization is widely recommended in functional and medical food research as a tool to facilitate the interpretation of complex sensory data.

### Limitations of the Study

A limitation of the present study is the use of a semi-trained sensory panel compo-sed of dietetics students, which may restrict the direct generalization of the obtained results to the clinical patient population. However, it should be emphasized that similar approaches are commonly applied in preliminary and comparative studies on food sensory evaluation, providing valuable information on the potential acceptability of pro-ducts prior to clinical testing.

Therefore, further research involving patients at risk of malnutrition appears to be justified. Future studies should also focus on evaluating the relationship between sensory acceptance and the actual consumption of foods for special medical purposes (FSMPs) under real clinical conditions.

## 5. Conclusions

The sensory evaluation revealed clear differences between the tested flavour variants. The highest overall sensory quality was observed for the white chocolate and raspberry, strawberry, and vanilla variants. These samples were characterized by a well-balanced flavour profile, high intensity of desirable sensory attributes, and a low presence of undesirable notes, which translated into the highest overall acceptability among participants.

In contrast, the neutral variant received the lowest scores across most evaluated parameters. This product was characterized by low flavour and aroma intensity combined with a relatively higher presence of undesirable notes, indicating limited sensory attractiveness and lower overall acceptance.

The coffee-flavoured variant demonstrated high aroma intensity; however, it was also associated with multiple sensory defects, including bitterness, metallic notes, graininess, and sediment presence. As a result, its overall sensory profile was perceived as less consistent and may require formulation optimization.

The findings suggest that both the neutral and coffee-flavoured variants may benefit from reformulation, particularly in terms of reducing undesirable sensory attributes and improving flavour balance. Consumer preference analysis further indicated that variants with a sweeter and more pronounced sensory profile were significantly more acceptable than neutral or coffee-flavoured products.

From a clinical perspective, these results are particularly relevant for foods for special medical purposes, as sensory acceptability is a key factor influencing patient adherence to nutritional recommendations and may directly affect the effectiveness of nutritional therapy.

## Figures and Tables

**Table 1:** Characteristics of powdered foods for special medical purposes (FSMPs) evaluated in the sensory analysis, including product line and flavour variants.

Product Line	Flavour Variant
Nutramil Complex Protein	Neutral
Nutramil Complex	White chocolate and raspberry
Nutramil Complex Protein	Vanilla
Nutramil Complex Protein	Strawberry
Immuven	Coffee

All products were powdered foods for special medical purposes manufactured by the same company (Olimp Laboratories, Poland).

**Table 2:** Nutritional composition of powdered foods for special medical purposes (FSMPs) evaluated in the sensory study (per 100 g of product). Own elaboration based on labels found on food products.

Product	Flavour	Energy (kcal)	Fats (g)	Saturated (g)	MCT ^1^ (g)	Carbohydrates (g)	Sugars (g)	Proteins (g)	Salt (g)
Olimp Nutramil Complex Protein	Neutral	417	15.3	6.9	5.7	46.0	5.6	26.9	0.6
Olimp Nutramil Complex	White chocolate/ raspberry	417	14.9	6.7	5.5	62.5	7.2 ^1^	15.7	0.60
Olimp Nutramil Complex Protein	Vanilla	417	14.9	6.7	5.5	44.7	5.4	26	0.59
Olimp Nutramil Complex Protein	Strawberry	417	14.9	6.7	5.5	44.7	5.4	26	0.59
Olimp Immuven	Coffee	365	10	0	1.9	50	5.5	23	0.19

^1^ MCT—Medium-chain fatty acids.

**Table 3:** Preparation procedure of powdered food for special medical purposes (FSMP) samples prior to sensory evaluation.

Trade Name of the FSMP Preparation	Taste	Method of Preparation
Olimp Nutramil Complex Protein	Neutral	Pour the contents of 1 sachet into 150 mL of boiled water at room temperature, stirring until completely dissolved. Consume immediately after preparation (200 mL of ready-made meal).
Olimp Nutramil Complex Protein	White chocolate/ raspberry	Pour the contents of 1 sachet into 170 mL of boiled water at room temperature, stirring until completely dissolved. Consume immediately after preparation (230 mL of ready-made meal).
Olimp Nutramil Complex Protein	Vanilla	Pour the contents of 1 sachet into 150 mL of boiled water at room temperature, stirring until completely dissolved. Consume immediately after preparation (200 mL of ready-made meal).
Olimp Nutramil Complex Protein	Strawberry	Pour the contents of 1 sachet into 150 mL of boiled water at room temperature, stirring until completely dissolved. Consume immediately after preparation (200 mL of ready-made meal).
Olimp Immuven	Coffee	Add 78 g (2 scoops) of powder to 150 mL of boiled water at room temperature, stirring until completely dissolved. Consume immediately after preparation (200 mL of the finished product).

**Table 4:** Sensory attributes and operational definitions used in the quantitative descriptive analysis (QDA) of FSMP products [14,15,16].

Sensory Attribute	Operational Definition
Colour intensity	Perceived strength or saturation of the product colour.
Colour uniformity	Degree of homogeneity in colour distribution throughout the prepared product.
Clarity	Degree of transparency or absence of turbidity in the reconstituted beverage.
Presence of sediment	Visible separation or deposition of particles after preparation.
Intensity of scent	Overall perceived strength of the aroma.
Natural aroma	Degree to which the aroma resembles expected natural flavour characteristics.
Purity of taste	Absence of foreign or off-flavour notes during taste perception.
Harmony of flavour	Balance and integration of flavour components.
Intensity of basic taste	Strength of the dominant basic taste (e.g., sweet).
Intensity of aftertaste	Persistence and strength of flavour perception after swallowing.
Undesirable notes (foreign/chemical)	Presence of artificial, chemical, or non-characteristic flavour impressions.
Undesirable notes (bitter/metallic)	Perception of bitterness or metallic taste.
Smoothness	Perceived absence of roughness during oral processing.
Creaminess	Perception of thickness and dairy-like mouthfeel.
Presence of lumps/graininess	Detection of undissolved particles during oral evaluation.

**Table 5:** Descriptive statistics of sensory attributes of five flavour variants of powdered foods for special medical purposes evaluated using quantitative descriptive analysis (QDA).

Sensory Characteristic	Product	N	($\bar{X}$)	SD	SE
Purity of taste	White chocolate and raspberry	49	5.306	2.426	0.347
	Coffee	49	4.531	2.093	0.299
	Neutral	49	2.408	2.791	0.399
	Strawberry	49	4.714	2.072	0.296
	Vanilla	49	5.265	2.168	0.310
Smoothness	White chocolate and raspberry	49	6.837	2.734	0.391
	Coffee	49	5.122	3.093	0.442
	Neutral	49	5.490	3.273	0.468
	Strawberry	49	6.388	2.871	0.410
	Vanilla	49	6.367	2.797	0.400
Harmony of flavour (are the ingredients well balanced?)	White chocolate and raspberry	49	5.816	2.759	0.394
	Coffee	49	4.122	2.862	0.409
	Neutral	49	1.429	2.000	0.286
	Strawberry	49	5.020	2.445	0.349
	Vanilla	49	5.551	2.492	0.356
Colour intensity	White chocolate and raspberry	49	4.694	1.884	0.269
	Coffee	49	4.837	1.546	0.221
	Neutral	49	4.204	2.021	0.289
	Strawberry	49	5.347	2.223	0.318
	Vanilla	49	5.041	1.979	0.283
Intensity of aftertaste	White chocolate and raspberry	49	6.633	2.472	0.353
	Coffee	49	7.224	2.285	0.326
	Neutral	49	4.122	3.251	0.464
	Strawberry	49	6.449	2.346	0.335
	Vanilla	49	5.571	2.102	0.300
Intensity of the basic flavour (e.g., sweet, salty)	White chocolate and raspberry	49	7.531	2.256	0.322
	Coffee	49	6.816	2.833	0.405
	Neutral	49	1.959	2.606	0.372
	Strawberry	49	6.490	2.526	0.361
	Vanilla	49	6.551	1.916	0.274
Intensity of the scent	White chocolate and raspberry	49	7.000	2.492	0.356
	Coffee	49	8.041	2.389	0.341
	Neutral	49	2.694	2.895	0.414
	Strawberry	49	6.694	2.600	0.371
	Vanilla	49	5.122	2.421	0.346
Colour uniformity	White chocolate and raspberry	49	5.857	2.111	0.302
	Coffee	49	3.735	1.934	0.276
	Neutral	49	6.469	2.416	0.345
	Strawberry	49	5.592	2.457	0.351
	Vanilla	49	6.469	2.328	0.333
Clarity (for liquid preparations)	White chocolate and raspberry	49	3.898	2.391	0.342
	Coffee	49	2.347	1.985	0.284
	Neutral	49	3.408	2.300	0.329
	Strawberry	49	3.816	2.195	0.314
	Vanilla	49	3.878	2.342	0.335
Creaminess	White chocolate and raspberry	49	6.061	2.066	0.295
	Coffee	49	5.163	2.528	0.361
	Neutral	49	5.327	2.794	0.399
	Strawberry	49	6.408	2.605	0.372
	Vanilla	49	6.429	2.189	0.313
Natural aroma	White chocolate and raspberry	49	4.653	2.743	0.392
	Coffee	49	6.143	2.475	0.354
	Neutral	49	2.041	2.685	0.384
	Strawberry	49	3.878	2.538	0.363
	Vanilla	49	4.041	2.423	0.346
Presence of lumps/graininess	White chocolate and raspberry	49	1.918	2.290	0.327
	Coffee	49	3.796	3.285	0.469
	Neutral	49	2.592	3.122	0.446
	Strawberry	49	2.265	2.456	0.351
	Vanilla	49	2.571	2.923	0.418
Presence of undesirable notes (e.g., foreign, chemical)	White chocolate and raspberry	49	3.592	3.214	0.459
	Coffee	49	2.898	2.974	0.425
	Neutral	49	4.102	3.938	0.563
	Strawberry	49	4.429	3.055	0.436
	Vanilla	49	3.265	2.430	0.347
Presence of undesirable notes (e.g., bitter, metallic)	White chocolate and raspberry	49	2.429	2.784	0.398
	Coffee	49	5.204	3.428	0.490
	Neutral	49	4.449	3.797	0.542
	Strawberry	49	3.367	2.604	0.372
	Vanilla	49	3.122	3.180	0.454
Presence of sediments or delamination	White chocolate and raspberry	49	5.306	2.763	0.395
	Coffee	49	6.776	2.710	0.387
	Neutral	49	4.980	2.780	0.397
	Strawberry	49	5.367	2.279	0.326
	Vanilla	49	5.204	2.685	0.384

N—sample size; ($\bar{x}$)—arithmetic mean; SD—standard deviation; SE—standard error.

**Table 6:** Results of Welch’s one-way ANOVA for sensory attributes of the evaluated FSMP flavour variants.

Sensory Characteristics	F	df1	df2	*p*
Purity of taste	9.637	4	119.686	<0.001
Smoothness	2.727	4	119.874	0.032
Harmony of flavour	31.912	4	119.449	<0.001
Colour intensity	2.000	4	119.487	0.099
Intensity of aftertaste	8.916	4	119.518	<0.001
Intensity of the basic flavour	36.955	4	119.374	<0.001
Intensity of the scent	28.793	4	119.874	<0.001
Colour uniformity	14.415	4	119.732	<0.001
Clarity	4.848	4	119.852	0.001
Creaminess	2.749	4	119.611	0.031
Natural aroma	15.795	4	119.935	<0.001
Presence of lumps/graininess	2.778	4	119.399	0.030
Presence of undesirable notes (e.g., foreign, chemical)	1.977	4	119.290	0.102
Presence of undesirable notes (e.g., bitter, metallic)	5.711	4	119.457	<0.001
Presence of sediments or delamination	3.334	4	119.801	0.013

F—value of the test statistic in analysis of variance (ANOVA); df1—degrees of freedom between groups; df2—degrees of freedom of error (adjusted, according to Welch’s test); *p*—level of statistical significance.

**Table 7:** Pairwise comparisons of sensory attributes between FSMP flavour variants based on post hoc analysis.

Attribute	Comparison	*p*-Value
Purity of taste	White chocolate and raspberry vs. coffee	0.467
Purity of taste	White chocolate and raspberry vs. neutral	<0.001
Purity of taste	White chocolate and raspberry vs. strawberry	0.716
Purity of taste	White chocolate and raspberry vs. vanilla	1.000
Purity of taste	Coffee vs. neutral	<0.001
Purity of taste	Strawberry vs. neutral	<0.001
Smoothness	White chocolate and raspberry vs. coffee	0.036
Harmony of flavour	White chocolate and raspberry vs. coffee	0.009
Harmony of flavour	White chocolate and raspberry vs. neutral	<0.001
Harmony of flavour	Coffee vs. neutral	<0.001
Harmony of flavour	Coffee vs. vanilla	0.044

*p*—level of statistical significance.

## Data Availability

The original contributions presented in this study are included in the article. Further inquiries can be directed to the corresponding author.

## Supplementary Materials

The following supporting information can be downloaded at: https://www.mdpi.com/article/10.3390/jcm15062188/s1.

## Abbreviations

The following abbreviations are used in this manuscript:

SBCIM	White chocolate and raspberry flavour
SK	Coffee flavour
SN	Neutral taste
ST	Strawberry flavour
SW	Vanilla flavour
