# Supplementary material for: Sensory Acceptability and Sensory Profiles of Flavoured Foods for Special Medical Purposes: A Quantitative Descriptive Analysis
Source: J Clin Med. 2026 Mar 13;15(6):2188. doi: 10.3390/jcm15062188 (PMC13027257; doi:10.3390/jcm15062188)
Supplement: Supplementary file 1 [file jcm-15-02188-s001.zip › Supplementary Materials S2.pdf]

KARTA OCENY SENSORYCZNEJ
ŻYWNOŚCI SPECJALNEGO PRZEZNACZENIA MEDYCZNEGO

Próbka ____________________________ **Data:** ___________ **Oceniający:** ________________________

Instrukcja oceny

Próbki oceniane są w losowej kolejności.
Przed każdą oceną należy oczyścić kubki smakowe wodą i bezcukrowym sucharem.
Oceny dokonuje się w skali od 0 do 10, gdzie:
0 = cecha niewyczuwalna
10 = cecha bardzo intensywna

---

1. Wygląd (wizualna ocena produktu)

Wyróżnik jakości	Ocena (0 - brak cechy, 10 - bardzo intensywna)
Jednolitość barwy	☐☐☐☐☐☐☐☐☐☐☐
Intensywność barwy	☐☐☐☐☐☐☐☐☐☐☐
Klarowność (dla płynnych preparatów)	☐☐☐☐☐☐☐☐☐☐☐
Obecność osadów lub rozwarstwień	☐☐☐☐☐☐☐☐☐☐☐

---

2. Zapach (ocena aromatu)

Wyróżnik jakości	Ocena (0 - brak cechy, 10 - bardzo intensywna)
Intensywność zapachu	☐☐☐☐☐☐☐☐☐☐☐
Naturalność aromatu	☐☐☐☐☐☐☐☐☐☐☐
Obecność niepożądanych nut (np. obcych, chemicznych)	☐☐☐☐☐☐☐☐☐☐☐

---

3. Smak

Wyróżnik jakości	Ocena (0 - brak cechy, 10 - bardzo intensywna)
Intensywność smaku podstawowego (np. słodki, słony)	☐☐☐☐☐☐☐☐☐☐☐
Harmonia smaku (czy składniki są dobrze zbalansowane?)	☐☐☐☐☐☐☐☐☐☐☐
Obecność nut niepożądanych (np. gorzki, metaliczny)	☐☐☐☐☐☐☐☐☐☐☐
Czystość smaku	☐☐☐☐☐☐☐☐☐☐☐
Intensywność posmaku	☐☐☐☐☐☐☐☐☐☐☐

---

4. Tekstura i konsystencja

Wyróżnik jakości	Ocena (0 - brak cechy, 10 - bardzo intensywna)
Gładkość	☐☐☐☐☐☐☐☐☐☐
Kremowość	☐☐☐☐☐☐☐☐☐☐
Obecność grudek / ziarnistość	☐☐☐☐☐☐☐☐☐☐

Uwagi oceniającego dotyczące przeprowadzonej oceny:

Słowniczek pojęć dotyczących tekstury w ocenie preparatów do żywienia medycznego:

✅ Gładkość – określa poziom jednolitości i braku wyczuwalnych cząstek stałych w preparacie. Produkt o wysokiej gładkości nie zawiera grudek, drobnych ziaren ani innych elementów zakłócających jednolitą strukturę. W preparatach medycznych gładkość jest kluczowa, szczególnie dla pacjentów z dysfagią (trudnościami w przełykaniu).

✅ Kremowość – odnosi się do odczucia bogatej, aksamitnej i pełnej konsystencji w ustach. Jest to cecha pożądana w preparatach wysokoenergetycznych, ponieważ wpływa na ich akceptowalność przez pacjentów. Preparat kremowy nie jest wodnisty ani zbyt gęsty, ale dobrze rozprowadza się po podniebieniu.

✅ Grudkowatość – obecność większych, nieregularnych cząstek, które mogą być wyczuwalne w jamie ustnej lub powodować nieprzyjemne wrażenie przy połykaniu.

✅ Ziarnistość – obecność bardzo drobnych, często jednorodnych cząstek, które nadają preparatowi szorstką strukturę. Może wynikać z niedokładnego rozpuszczenia składników lub użycia nierafinowanych surowców.
