# Supplementary material for: Sensory Acceptability and Sensory Profiles of Flavoured Foods for Special Medical Purposes: A Quantitative Descriptive Analysis
Source: J Clin Med. 2026 Mar 13;15(6):2188. doi: 10.3390/jcm15062188 (PMC13027257; doi:10.3390/jcm15062188)

Radar Sensory Profile of the White Chocolate and Raspberry Variant (QDA)

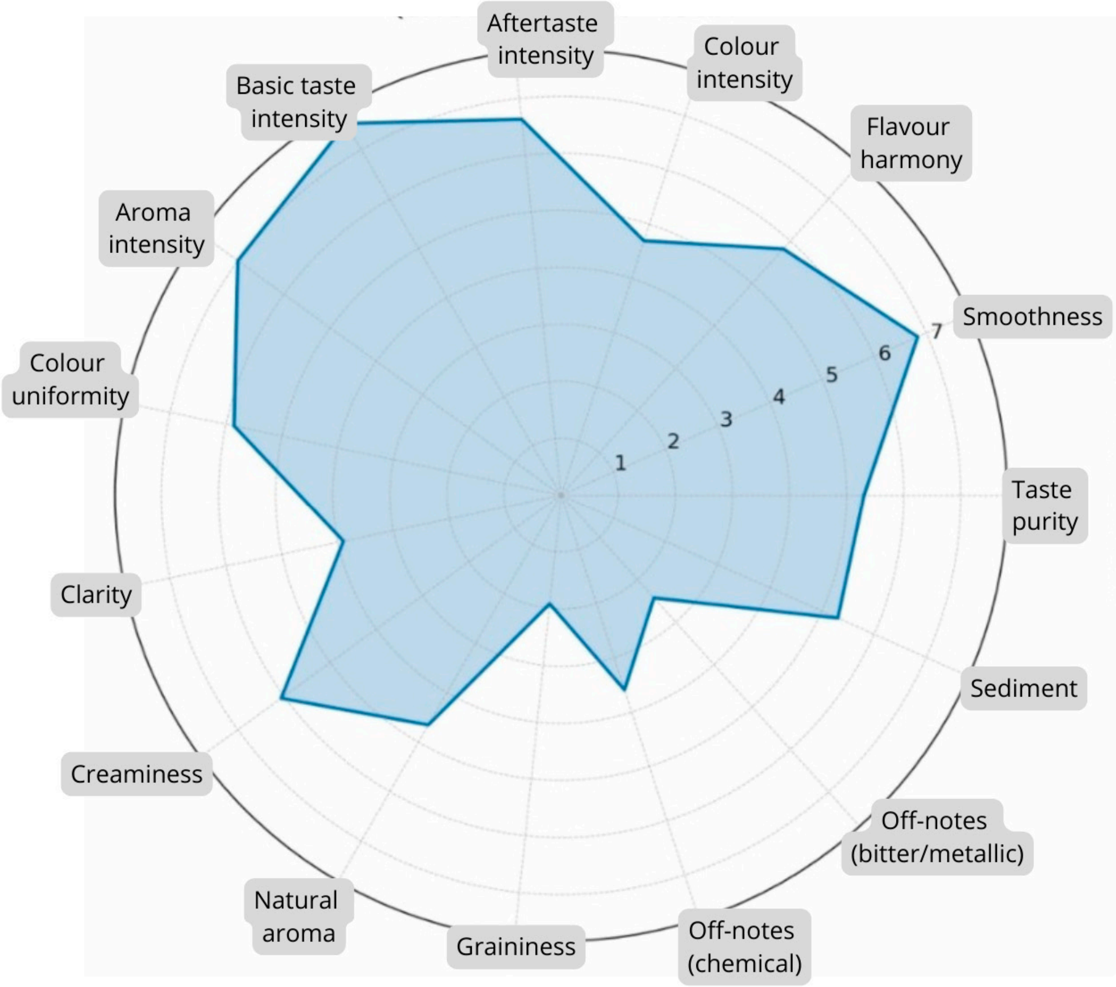

Radar Sensory Profile of the Coffee (QDA)

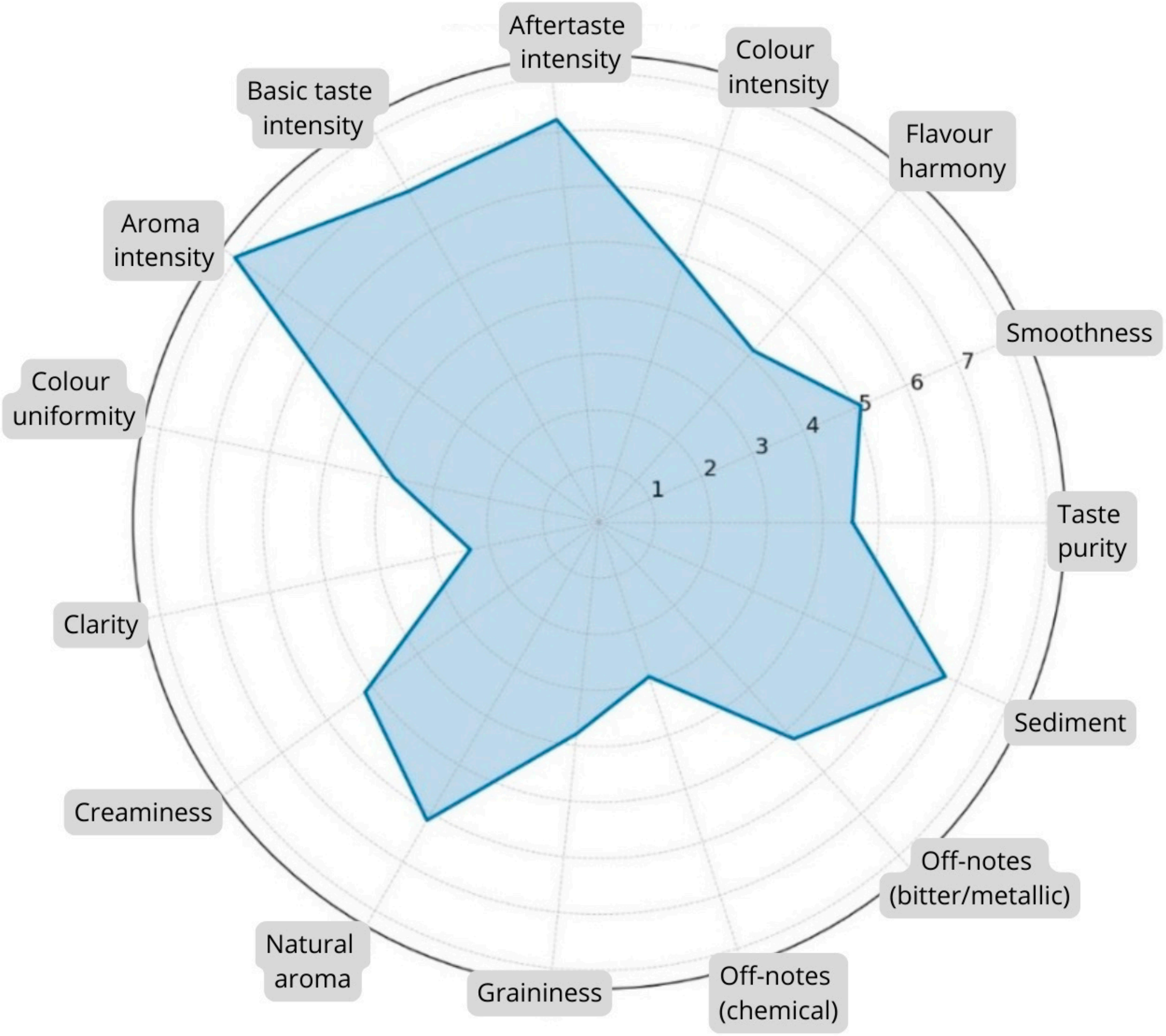

Radar Sensory Profile of the Neutral (QDA)

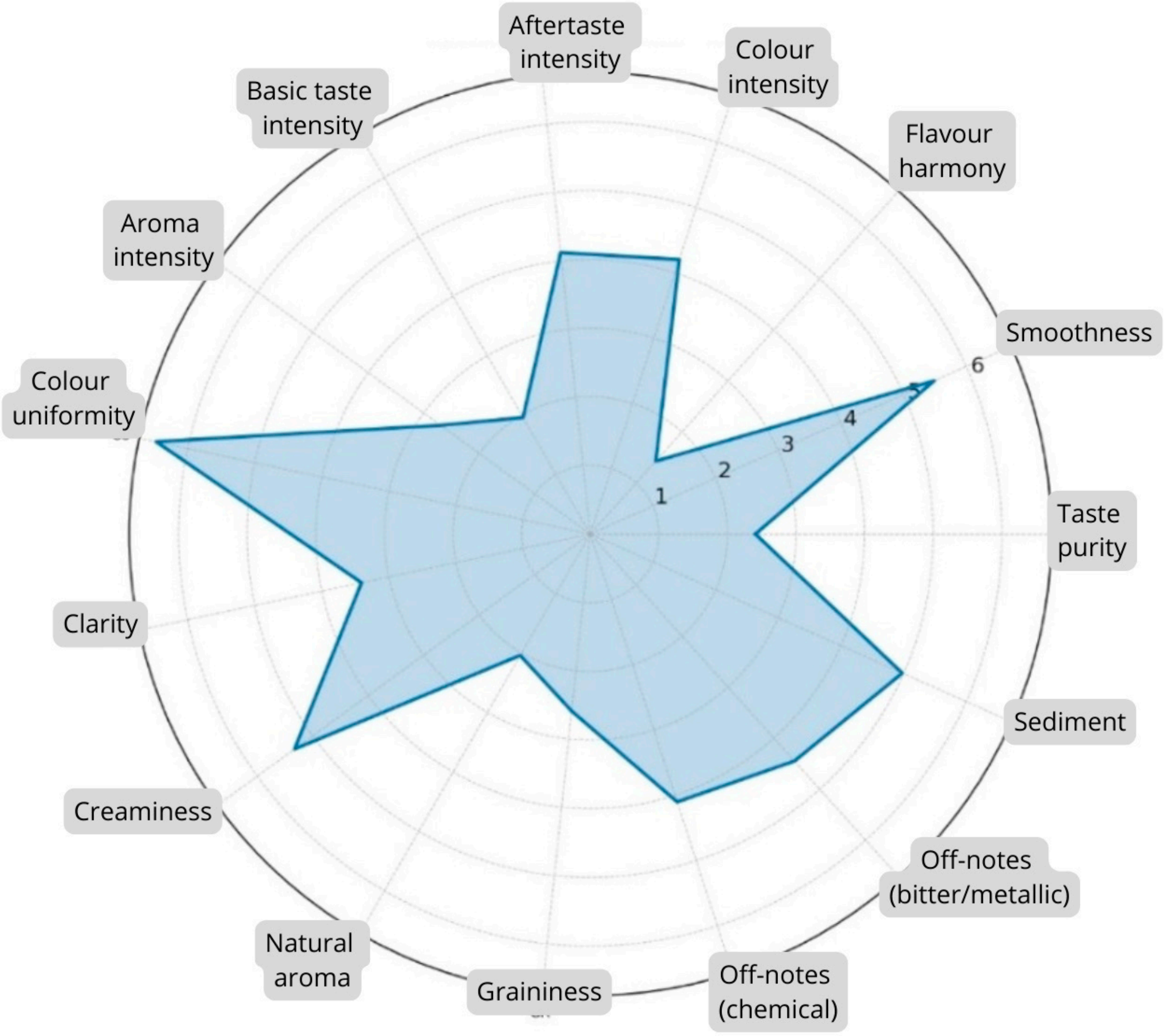

Radar Sensory Profile of the Strawberry (QDA)

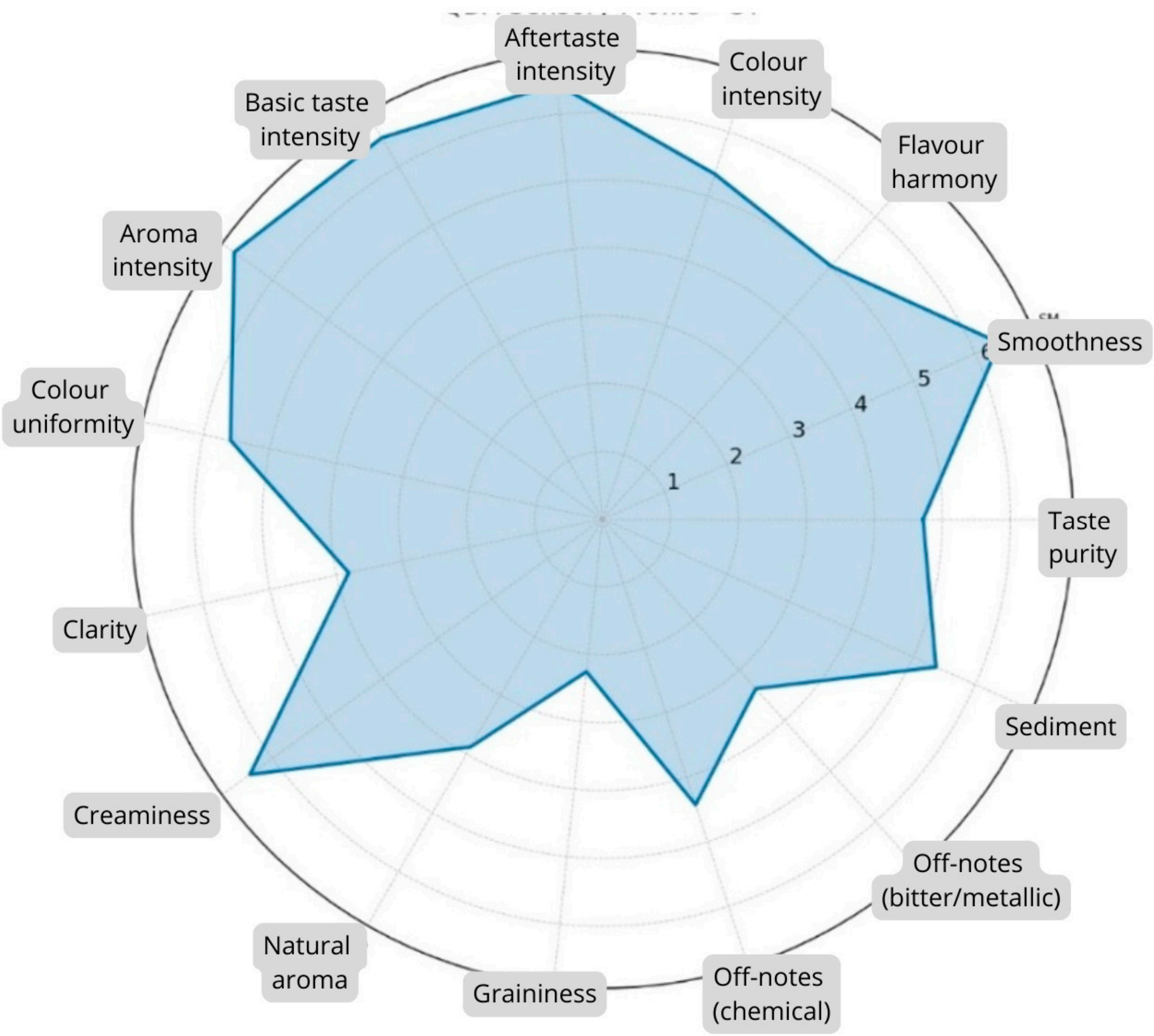

Radar Sensory Profile of the Vanilla (QDA)

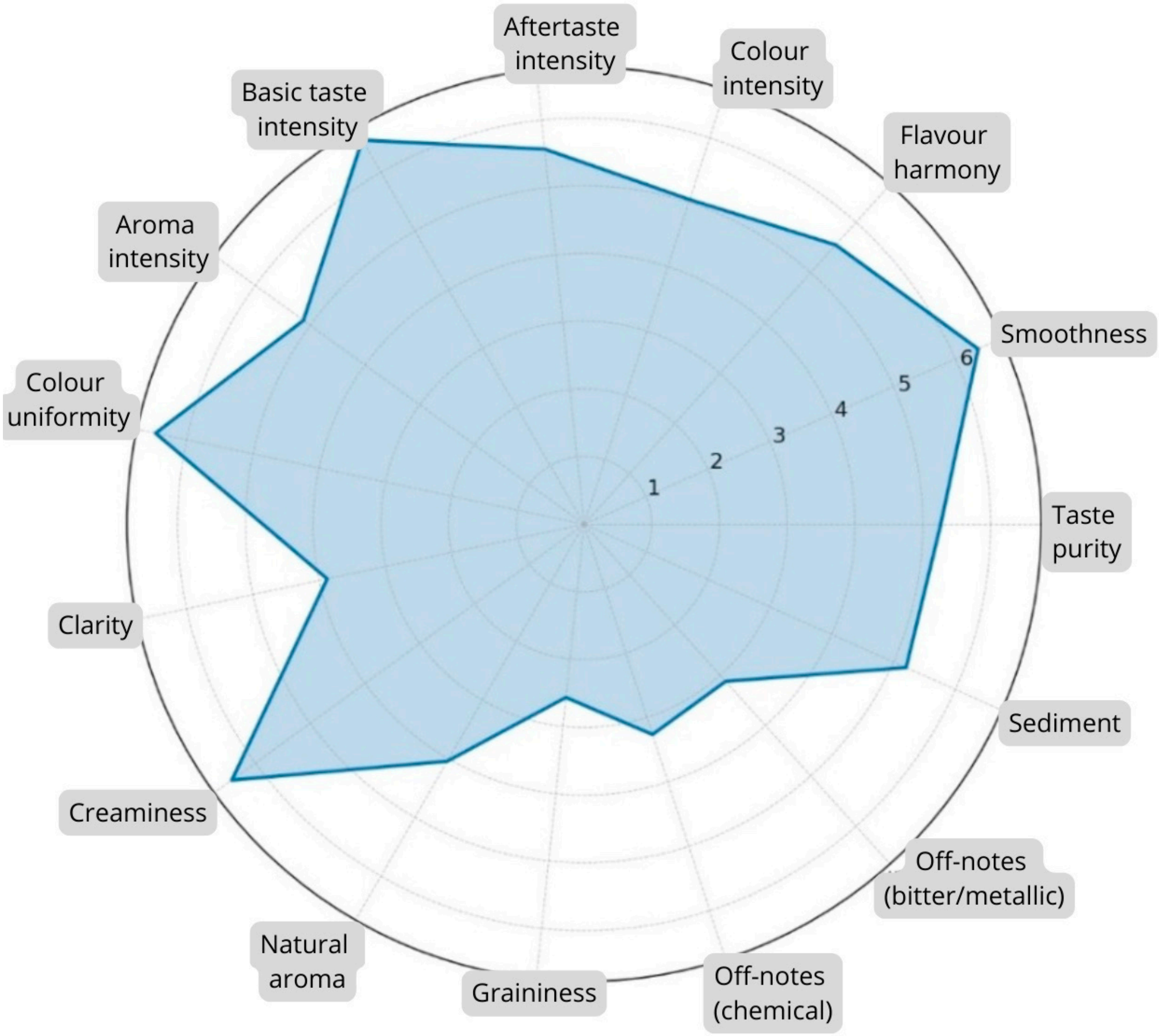

Supplement: Supplementary file 1 [file jcm-15-02188-s001.zip › Supplementary Materials S1.pdf]
